# Genetic polymorphism in *BIN1* rather than *APOE* is associated with poor recognition memory among men without dementia

**DOI:** 10.1038/s41598-022-20587-9

**Published:** 2022-10-24

**Authors:** Kanika Mehta, Mohammadreza Mohebbi, Julie A. Pasco, Lana J. Williams, Ken Walder, Boon Lung Ng, Veer Bala Gupta

**Affiliations:** 1 Deakin University, IMPACT – The Institute for Mental and Physical Health and Clinical Translation, School of Medicine, Geelong, VIC Australia; 2 Biostatistics Unit, Faculty of Health, Deakin University, Burwood, VIC Australia; 3 Department of Medicine-Western Health, The University of Melbourne, St Albans, VIC, Australia; 4 Department of Epidemiology and Preventive Medicine, Monash University, Prahran, VIC Australia; 5 Barwon Health, Geelong, VIC Australia; 6 Department of Geriatric Medicine, Barwon Health, Geelong, VIC Australia

**Keywords:** Genetics, Neuroscience, Biomarkers, Risk factors

## Abstract

Although several genetic polymorphisms have been linked with the risk of Alzheimer’s disease, less is known about their impact on cognitive performance among cognitively healthy individuals. Our aim was to investigate the association of the genetic variant, rs744373 in the bridging integrator 1 gene (*BIN1*), the strongest genetic risk factor for Alzheimer’s disease after the *APOE* ε4 allele, with different cognitive domains among non-demented older men. Cognitive function was measured using the CogState Brief Battery, which assessed cognitive performance across four domains: psychomotor function, visual attention, recognition memory and working memory. Linear regression analysis revealed that individuals with the *BIN1* risk allele performed poorly on the recognition memory task as compared to those without the risk allele. However, this was in contrast with the individuals who harboured the *APOE* ε4 risk allele as they displayed better performance on the recognition task in comparison to those without the ε4 risk allele. To the best of our knowledge, this is the first study that demonstrates genetic variation in *BIN1* to be a better predictor of recognition memory than *APOE*, which remains the biggest genetic risk factor for Alzheimer’s disease.

## Introduction

Late-onset Alzheimer’s disease (LOAD) is a multifactorial disease that involves an interplay between several genetic and environmental factors. Despite this, research is mainly focused on the role of the *APOE* ε4 allele towards the presentation of LOAD, as this remains the most studied genetic risk factor to date. Interestingly, LOAD can also develop among individuals without the *APOE* risk allele, highlighting the need to investigate other risk-conferring genetic polymorphisms. Large genome-wide association studies (GWAS) have identified several susceptibility genes/loci linked to high risk of LOAD. Among them, the single nucleotide polymorphism (SNP) rs744373 in the bridging integrator 1 (*BIN1*) gene has displayed the highest effect size for LOAD, second only to the *APOE* ε4 allele^1,2^. The global frequency of the G allele (risk allele) is 37% and individuals harbouring the risk allele have at least 1.17 higher odds of developing LOAD according to several reports including meta-analyses^3–7^. The locus rs744373 is located within 30 kb upstream of the coding region, which encodes for the protein Amphiphysin 2^1,8^. It belongs to a family of BIN1/Amphiphysin/RVS167 (BAR) adaptor proteins that are involved in the regulation of lipid membrane dynamics^9^.

The association of *BIN1* rs744373 with LOAD has been replicated across different ethnic populations^6,10^; however, its role in mediating LOAD risk remains uncertain. As Alzheimer’s disease (AD) is believed to be preceded by a long preclinical phase, it is important to unravel the association of *BIN1* with cognitive function among non-demented individuals as this may provide evidence of the role *BIN1* has in the development of AD. A previous study conducted on a young Chinese population revealed that cognitively normal individuals who were homozygous for the rs744373 allele had worse working memory performance and lower functional connectivity in comparison to their non-carrier counterparts, highlighting *BIN1*’s role in early cognitive changes^11^. However, the impact of *BIN1* on multiple cognitive domains has not been explored in large population-based cohorts, highlighting a gap in our understanding of its risk profile. Hence, the current study investigates the association between the *BIN1* rs744373 SNP and performance across different cognitive domains, and compares these findings with *APOE* ε4 allele, the most established risk factor for AD, among healthy ageing men free of severe cognitive impairment or dementia.

## Material and methods

### Study cohort

The present study analysed data and blood samples collected from men recruited as a part of the Geelong Osteoporosis Study (GOS), an ongoing prospective population-based study. In brief, age-stratified samples of men and women were selected at random from electoral rolls for the Barwon Statistical Division in south-eastern Australia^12^. A total of 1,540 men were recruited at the baseline from 2001 to 2006 (67% participation), followed by 5-, 6- and 15-year re-assessment phases. This study includes a cross-sectional analysis of data and blood samples collected from 449 men during the 15-year follow-up phase (2016–2020). Participants were mostly Caucasian (~ 98%). They provided information on their lifestyle and demographic characteristics in addition to undergoing mental and physical health assessments. Inclusion criteria were a listing on the electoral rolls for the Barwon Statistical Division and residence in the area for a minimum of 6 months. All participants provided written informed consent to participate in the study, which was approved by the Human Research Ethics Committee at Barwon Health. All procedures performed were in accordance with the ethical standards of the institutional and national research committees and with the 1964 Helsinki declaration and its later amendments or comparable ethical standards.

### Assessment procedures and sample collection

Cognitive function was evaluated using a computer-based neuropsychology battery, the CogState Brief Battery (CBB), which has been described previously^13–15^. The CBB requires participants to respond to stimuli cards as a part of a detection (DET), identification (IDN), one-card learning (OCL) and one-back (OBK) task that assessed cognitive performance across four domains, namely psychomotor function, visual identification/attention, recognition memory/learning and working memory, respectively. Both a practice trial and a real test were included for each task. The tasks were completed by participants in a quiet room accompanied by a researcher. For the tasks DET, IDN and OBK, scores were calculated by measuring the time (milliseconds) taken to answer correctly, which was then normalised using a log_10_ transformation. For the OCL task, scores were calculated based on the accuracy of participant response and normalised using an arcsine square-root transformation. Further, scores for the overall cognitive function (OCF) were determined by combining the primary measures in the four domains. Thus, for the tasks DET, IDN and OBK, lower scores suggested better cognitive performance and for OCL and OCF, higher scores indicated better performance. The individual scores on the four tasks and composite scores were utilised in the present analysis. In addition, participants underwent the Mini-Mental State Examination (MMSE), which assessed their overall cognitive function^16^.

### Other measures

Details on sociodemographic variables such as education, smoking and marital status were acquired from self-reports. Education was defined as a nominal factor based on secondary education completion. Similarly, marital status (living with a partner) was defined as living with a partner (coded “1”) or not (coded “0”). Participants who reported smoking at least one cigarette per day were defined as current smokers. The Structured Clinical Interview for *Diagnostic and Statistical Manual of Mental Disorders, Fourth Edition,* Non-Patient Edition (SCID-I/NP) was used to determine a lifetime history of mood disorders, as described previously^17^.

### DNA extraction and genotyping

Blood collected from participants after overnight fasting was separated into different aliquots of serum, plasma and buffy coats, and stored at − 80 °C until use. Total genomic DNA was isolated from buffy coats using QIAamp® DNA Mini Kit (Qiagen, Hilden, Germany) as per the manufacturer’s instructions. The DNA samples were genotyped for the SNPs rs429358 (*APOE* ε4), rs7412 (*APOE* ε2) and rs744373 (*BIN1*) at the Australian Genome Research Facility, Brisbane using the Agena Bioscience MassARRAY® platform. The carrier status was defined by the presence of at least one copy of the risk allele. Hence, in the present study GG/GA and AA genotypes were referred as *BIN1* G+ and *BIN1* G−, respectively. Similarly, *APOE* ε4 + referred to the presence of at least one ε4 allele. The allelic distribution for both *BIN1* and *APOE* did not depart from the Hardy–Weinberg equilibrium.

### Statistical analyses

Characteristics were compared across *BIN1* G+ and *BIN1* G−, and *APOE* ε4+ and *APOE* ε4- participants using Student's t-tests for continuous variables and chi-squared tests for categorical variables. Simple linear regression analyses were conducted to investigate the association between *BIN1* carrier status and cognitive function. The outcome, cognitive function included scores on each of the four tasks and OCF. Further, multivariable linear regression models adjusted for age and *APOE* carrier status were developed for each outcome. Similarly, unadjusted and age-adjusted linear regression analyses were conducted with *APOE* status as the exposure variable and cognitive function as the outcome. Following this, interactions between *BIN1* and *APOE* risk alleles were explored using unadjusted and age-adjusted regression models for all five outcomes. Finally, the association between *BIN1* carrier status and cognitive function was compared among *APOE* ε4 carriers and non-carriers to investigate whether the effect of *BIN1* differs between the two groups. Benjamini–Hochberg correction was applied to adjust for false discovery rate due to multiple testing^18^. All statistical analyses were performed using Stata/SE 17.0 and Python 3.8.5.

## Results

### Participant characteristics and intergroup differences

Participant characteristics are presented in Table 1. The study participants had a mean age of 64.3 years (SD 13.3) and roughly three quarters had completed secondary education (75.2%). No significant differences were observed between groups stratified by *BIN1* status. When stratified by *APOE* ε4 status, ε4 carriers were found to be younger than non-carriers (*p* < 0.01) and had a slightly higher MMSE score (*p* 0.024).

**Table 1:** Demographic characteristics of the study participants. Data are presented as mean (SD) or n(%).

	Overall n = 449	*BIN1* G+ n = 229	*BIN1* G− n = 220	*p*-value	Overall n = 445	*APOE* ε4 + n = 116	*APOE* ε4− n = 329	*p*-value
**Age [years], mean (SD)**	64.3 (13.3)	64.9 (14.4)	63.7 (11.9)	0.328	64.2 (13.3)	61.0 (13.7)	65.4 (13.0)	< 0.01
**Education, n (%)**								
Secondary education completed	337 (75.2)	165 (72.4)	172 (78.2)	0.154	334 (75.2)	94 (81.7)	240 (72.9)	0.060
Secondary education not completed	111 (24.8)	63 (27.6)	48 (21.8)		110 (24.8)	21 (18.3)	89 (27.1)	
**Marital status (living with a partner), n (%)**								
Living with a partner	364 (81.1)	184 (80.3)	180 (81.8)	0.691	361 (81.1)	94 (81.0)	267 (81.2)	0.977
Not living with a partner	85 (18.9)	45 (19.7)	40 (18.2)		84 (18.9)	22 (19.0)	62 (18.8)	
**Current smoker, n (%)**								
Yes	31 (6.9)	18 (7.9)	13 (5.9)	0.415	31 (7.0)	7 (6.0)	24 (7.3)	0.647
No	418 (93.1)	211 (92.1)	207 (94.1)		414 (93.0)	109 (94.0)	305 (92.7)	
***APOE* ε4 carriage, n (%)**								
Yes	116 (26.1)	60 (26.5)	56 (25.6)	0.814				
No	329 (73.9)	166 (73.5)	163 (74.4)					
**Lifetime history of a mood disorder, n (%)**								
Yes	107 (23.8)	55 (24.0)	52 (23.6)	0.925	105 (23.6)	32 (27.6)	73 (22.2)	0.239
No	342 (76.2)	174 (76.0)	168 (76.4)		340 (76.4)	84 (72.4)	256 (77.8)	
**MMSE, mean (SD)**	28.6 (1.7)	28.4 (1.9)	28.8 (1.5)	0.049	28.6 (1.7)	28.9 (1.4)	28.5 (1.8)	0.024

### Association of *BIN1* and *APOE* with the cognitive function

Table 2 shows results from the linear regression analyses for the association between *BIN1* carrier status and cognitive function. *BIN1* carrier status was associated with OCL (B_coeff_ − 0.03 95% CI [− 0.05, − 0.01], *p* < 0.01), suggesting that individuals with the risk allele had lower scores for OCL and displayed poorer performances on the learning task. Age and *APOE* status had no effect on the association between *BIN1* and OCL. Thus, the average OCL scores were 0.03 units lower for individuals with the *BIN1* risk allele, independent of age and *APOE* status. The association remained significant after the Benjamini–Hochberg correction. *BIN1* also showed a trend for inverse association with the overall cognitive function that was approaching statistical significance (B_coeff_ − 0.11 95% CI [− 0.23, 0.01], *p* 0.076).

**Table 2:** Linear regression analyses for predicting cognitive function using *BIN1* carrier status. IDN (identification) task measures visual identification/attention, DET (detection) task measures psychomotor function, OCL (one-card learning) task measures recognition memory/learning, OBK (one-back) task measures working memory and OCF refers to the overall cognitive function.

Outcome	Unadjusted model			Age and *APOE* adjusted model		
	B_coeff_ (95% CI)	*p*-value	Partial eta-squared	B_coeff_ (95% CI)	*p*-value	Partial eta-squared
IDN	0.002 (− 0.013, 0.016)	0.808	< 0.01	− 0.0004 (− 0.0134, 0.0125)	0.950	< 0.01
DET	0.01 (− 0.01, 0.03)	0.276	< 0.01	0.01 (− 0.01, 0.03)	0.428	< 0.01
OCL	− 0.03 (− 0.05, − 0.01)	< 0.01	0.03	− 0.03 (− 0.05, − 0.01)	< 0.01*	0.03
OBK	0.01 (− 0.01, 0.03)	0.210	< 0.01	0.01 (− 0.01, 0.03)	0.310	< 0.01
OCF	− 0.137 (− 0.276, 0.002)	0.054	0.01	− 0.11 (− 0.23, 0.01)	0.076	0.01

*Significant after false discovery rate (Benjamini–Hochberg) correction.

When different cognitive function outcomes were regressed over *APOE* status in the unadjusted model, a significant association was observed for the outcome OCL (B_coeff_ 0.03 95% CI [0.01, 0.05], *p* < 0.01) as shown in Table 3. However, this positive association indicated that individuals with the *APOE* ε4 allele performed better on the learning task and their average scores were 0.03 units higher. Similar results were obtained following adjustment for age. This contrasted with the results obtained for *BIN1*, which displayed a negative association with cognitive performance on the learning task.

**Table 3:** Linear regression analyses for predicting cognitive function using *APOE* carrier status. IDN (identification) task measures visual identification/attention, DET (detection) task measures psychomotor function, OCL (one-card learning) task measures recognition memory/learning, OBK (one-back) task measures working memory and OCF refers to the overall cognitive function.

Outcome	Unadjusted model			Age-adjusted model		
	B_coeff_ (95% CI)	*p*-value	Partial eta-squared	B_coeff_ (95% CI)	*p*-value	Partial eta-squared
IDN	0.01 (− 0.01, 0.02)	0.306	< 0.01	0.019 (0.005, 0.034)	0.011*	0.01
DET	− 0.01 (− 0.03, 0.02)	0.601	< 0.01	0.01 (− 0.02, 0.03)	0.560	< 0.01
OCL	0.03 (0.01, 0.05)	< 0.01	0.02	0.024 (0.003, 0.044)	0.023*	0.01
OBK	0.01 (− 0.02, 0.03)	0.510	< 0.01	0.025 (0.004, 0.046)	0.020*	0.01
OCF	0.05 (− 0.11, 0.21)	0.544	< 0.01	− 0.08 (− 0.21, 0.06)	0.277	< 0.01

*Significant after false discovery rate (Benjamini–Hochberg) correction.

**Figure 1:**
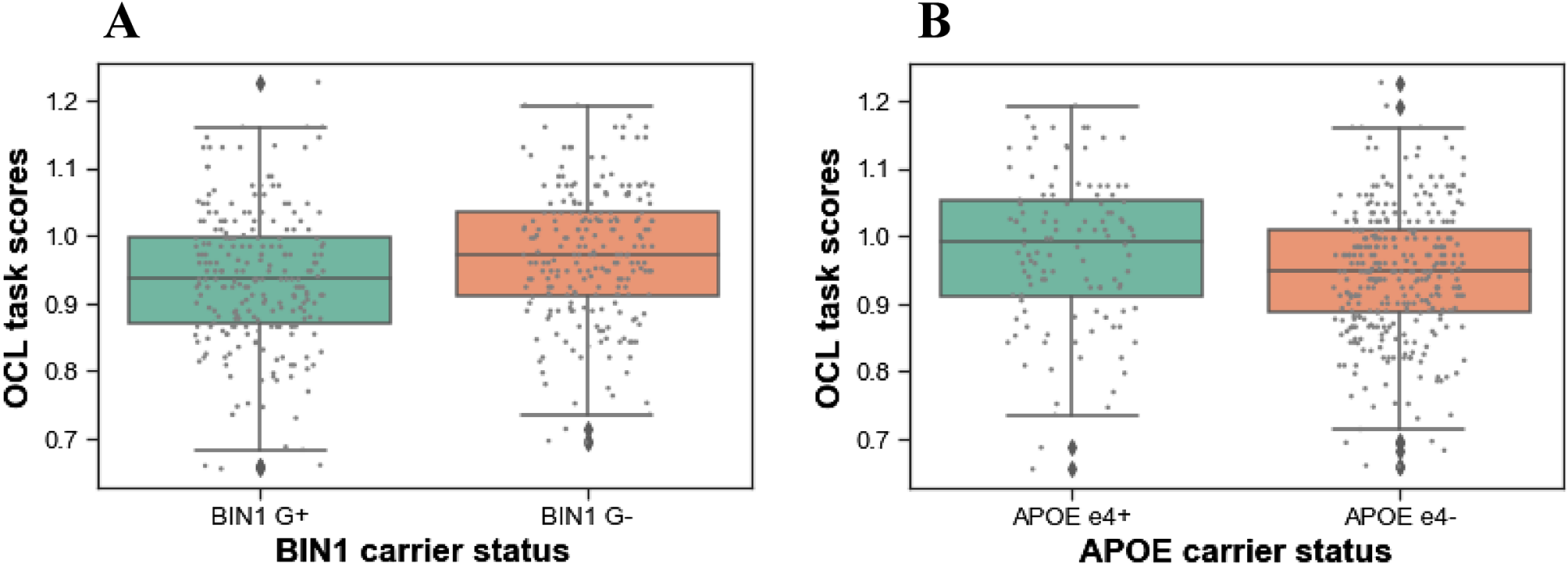
Boxplot depicting a comparison of the OCL task scores. (**A**) Among *BIN1* risk allele carriers and non-carriers. (**B**) Among *APOE* risk allele carriers and non-carriers.

Figure 1 outlines the comparison of cognitive scores on the OCL task (A) among *BIN1* carriers and non-carriers, and (B) among *APOE* carriers and non-carriers. In the age-adjusted model of *APOE*, significant positive associations were also observed for the outcomes IDN (B_coeff_ 0.019 95% CI [0.005, 0.034], *p* 0.011) and OBK (B_coeff_ 0.025 95% CI [0.004, 0.046], *p* 0.020), suggesting that the *APOE* ε4 allele is associated with poorer performance on the visual attention and working memory tasks, respectively. These results survived the Benjamini–Hochberg correction.

### Association of *BIN1* with cognitive function among *APOE* ε4 carriers and non-carriers

When study participants were stratified according to *APOE* status, *BIN1* showed significant negative associations with the OCL domain among both ε4 carriers (B_coeff_ − 0.05 95% CI [− 0.09, − 0.01], *p* < 0.01) and non-carriers (B_coeff_ − 0.024 95% CI [− 0.043, − 0.004], *p* 0.019), although a higher effect size was observed for the former, which also survived the Benjamini–Hochberg correction (Table 4). In addition, *BIN1* was also significantly associated with overall cognitive function among *APOE* ε4 carriers (B_coeff_ − 0.27 95% CI [− 0.50, − 0.03], *p* 0.027); however, no significant association was detected among non-carriers.

**Table 4:** Association of *BIN1* with cognitive function among *APOE* carriers and non-carriers. IDN (identification) task measures visual identification/attention, DET (detection) task measures psychomotor function, OCL (one-card learning) task measures recognition memory/learning, OBK (one-back) task measures working memory and OCF refers to the overall cognitive function.

Outcome	*APOE* ε4 non-carriers			*APOE* ε4 carriers		
	B_coeff_ (95%CI)	*p*-value	Partial eta-squared	B_coeff_ (95%CI)	*p*-value	Partial eta-squared
IDN	− 0.01 (− 0.02, 0.01)	0.420	< 0.01	0.02 (− 0.01, 0.04)	0.261	0.01
DET	0.001 (− 0.023, 0.024)	0.948	< 0.01	0.026 (− 0.003, 0.056)	0.081	0.03
OCL	− 0.024 (− 0.043, − 0.004)	0.019	0.02	− 0.05 (− 0.09, − 0.01)	< 0.01*	0.06
OBK	0.01 (− 0.01, 0.03)	0.431	< 0.01	0.01 (− 0.02, 0.05)	0.480	< 0.01
OCF	− 0.05 (− 0.19, 0.09)	0.456	< 0.01	− 0.27 (− 0.50, − 0.03)	0.027	0.04

*Significant after false discovery rate (Benjamini–Hochberg) correction.

Interaction between *BIN1* and *APOE* was also explored; however, it was not found to be statistically significant (results provided in Supplementary Table 1).

## Discussion

In this study, we examined cross-sectional associations between *BIN1*/*APOE* and cognitive function in non-demented men. In both the unadjusted and adjusted models, *BIN1* was inversely associated with cognitive performance on the OCL task that assessed recognition memory. This resonates with a previous study where healthy individuals, homozygous for the rs744373 allele, displayed worse working memory performance, larger hippocampal volume and lower functional connectivity^11^. In another study comprising healthy controls and participants with mild cognitive impairment (MCI), the risk allele was associated with worse memory performance, which was also mediated via elevated global tau levels^3^. Although the rs744373 allele has been identified as the second strongest genetic risk factor for LOAD only next to *APOE*, its mechanistic link with AD remains uncertain. A faster tau accumulation has been previously observed among *BIN1* G + participants and hence its role in modulating tau pathology has been ascribed as the biggest contribution to AD^19,20^. Further, knockout mice models of *BIN1* have revealed that the loss of *Bin1* neuronal expression results in the impairment of spatial learning and memory, highlighting its role in memory function^8^. However, it is still not clear whether the risk allele is associated with early cognitive development or cognitive decline in later life. A study by Glennon et al*.* found that the BIN1 protein alterations in human brain tissue are associated with the pathogenesis of sporadic but not familial AD^21^. The authors further suggested that the alterations in BIN1 protein levels are not associated with AD neurodegeneration or the ageing process^21^. A possible explanation could be long-standing differences in cognitive development among carriers and non-carriers causing the former to show cognitive deterioration sooner.

Our findings also revealed the *APOE* ε4 allele to be associated with lower scores on the IDN and OBK tasks but higher scores on the OCL task. Having been identified as the strongest genetic risk factor for LOAD, *APOE* has been linked to poor cognitive function on numerous occasions and thus its association with better recognition memory appeared counterintuitive. However, a recent study comprising 398 cognitively normal individuals aged ~ 70 years revealed that carriers of the ε4 risk allele performed better on the visual working memory task as compared to the non-carriers^22^. The authors argued that the *APOE* gene might be an example of antagonistic pleiotropy, conferring both beneficial and deleterious effects; therefore, contributing to the survival of this gene^22^. In another study, an age-based differential impact of *APOE* was observed on verbal memory performance, supporting the hypothesis of antagonistic pleiotropy^23^. Among individuals less than 57 years, the *APOE* ε4 allele was associated with verbal memory improvement, whereas ε4 carriers above 57 years displayed a decline in verbal memory^23^. This adds to the growing body of evidence that suggests *APOE* to exert a protective effect at a younger age. In addition to age, sex may also influence *APOE*’s relationship with cognitive performance as suggested by Zokaei and co-workers who found middle-aged males with the *APOE* ε4 allele to have a cognitive advantage on the memory task^24^. This beneficial effect of the *APOE* ε4 allele was not observed among women^24^ who historically have been at a higher risk of developing AD^25^.

We only observed significant associations between risk alleles and cognitive function; however, no significant interaction was detected between them. This could probably be due to a small effect size or sample size. Hence, we conducted an exploratory subgroup analysis that compared the association of *BIN1* with cognitive function among *APOE* carriers and non-carriers. We found *BIN1* to be negatively associated with recognition memory, regardless of *APOE* status, although a greater effect size was observed for the *APOE* ε4 carriers. In addition, *BIN1* was also found to be significantly associated with overall cognitive function among the ε4 carriers.

It is interesting to note that in our study *BIN1* showed associations primarily with recognition memory and not with other cognitive domains. However, this was an exploratory study that needs to be replicated across larger cohorts with a longitudinal study design. It would be worthwhile to investigate whether any differences exist between *APOE* and *BIN1* in predicting long-term change for different cognitive domains. Therefore, a major limitation of our study was the use of cross-sectional data only for men. However, we are collecting similar data for women. In addition, the population was mostly Caucasian and hence the findings may not be generalisable to other populations. Some of the strengths of our study include a population-based cohort as participants were drawn at random from the general population and were not selected on the basis of disease. Also, individuals with severe cognitive impairment or dementia were excluded from the study.

Overall, our study suggests that *BIN1* may be a better indicator of poor recognition memory than *APOE* in non-demented older men. In light of the above evidence, it is important to investigate the effect of genetic risk factors other than *APOE* on different cognitive domains and their biological function in the brain as this may improve our understanding of the pathophysiology of AD and provide novel therapeutic targets. The underlying role of these genes in AD pathogenesis may be different, and as a result, their impact on different cognitive domains among non-demented individuals may vary. Furthermore, the interaction between genetic risk factors and sex in modulating cognitive performances remains an area worth investigating. It would be interesting to see whether sex modifies *BIN1*’s association with memory function. Therefore, future prospective studies are required to further evaluate these findings along with brain imaging information in order to correlate them with the Aβ and tau pathology.

## Supplementary Information

Supplementary Tables.

## Data Availability

The genetic data analysed in this study are provided in Supplementary Table 2.
